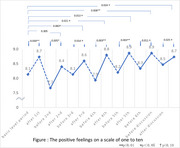# The effect of reminiscence using maps application positive feeling evaluated oneself

**DOI:** 10.1002/alz70860_096836

**Published:** 2025-12-23

**Authors:** Ayame Katayama

**Affiliations:** ^1^ Naragakuen University, Nara, Nara, Japan

## Abstract

**Background:**

The problem regarding dementia is increasing. Accordingly, reminiscence methods are attracting attention as a prevention of dementia. In addition, virtual reality in the medical field has been attracting attention. But reminiscence using VR research has not yet been generalized because the number of research subjects is a small number of people. The purpose of this research is the effect of reminiscence using maps application in VR space for positive feeling of community‐dwelling elderly to reveal the potential for dementia prevention.

**Method:**

This research is quasi experimental research. The reminiscence is conducted once a week for 40 minutes for a total of 6 weeks, using VR for 15 minutes out of the 40 minutes. The subjects are elderly people living in the community who are cognitively healthy. Positive feelings are evaluated by themselves on a 10‐point scale. The evaluation periods were 14 times in total: the basic level period, after the first recollection, before and after the second to sixth recollection, and before and after the discussion (the discussion one month after the completion of all the recollection periods). Analysis was conducted using IBM SPSS Ver.26 with Wilcoxon's signed rank test with a correspondence sample. The significance level is set at 5%. This study was approved by the Graduate School Research Ethics Review Committee of Naragakuen University (院 5–004).

**Result:**

Fifteen subjects were included in the study, excluding two subjects with insufficient data. The mean change in positive feelings on a scale of one to ten is shown in the figure. There was a significant increase at the six times before and after each reminiscence and before and after the discussion. Comparison of the data from the baseline period and after each reminiscence showed an upward trend or significant increase, except for the second session.

**Conclusion:**

Reminiscence using the MAPS application, when performed continuously and multiple times at intervals of one to two weeks, maintained and revitalized the positive emotions of the study participants. It is suggested that this may prevent depressive mood swings and depression, thereby preventing dementia.